# BmRRS1 Protein Inhibits the Proliferation of Baculovirus *Autographa californica* Nucleopolyhedrovirus in Silkworm, *Bombyx mori*

**DOI:** 10.3390/ijms25010306

**Published:** 2023-12-25

**Authors:** Liqin Zhou, Xinyi Ding, Zhisheng Wang, Si Zhou, Sheng Qin, Xia Sun, Xueyang Wang, Muwang Li

**Affiliations:** 1 Jiangsu Key Laboratory of Sericultural Biology and Biotechnology, School of Biotechnology, Jiangsu University of Science and Technology, Zhenjiang 212100, China; 18896659474@163.com (L.Z.); ding_xin_yi@163.com (X.D.); liexuewang1995@gmail.com (Z.W.); zhousi20220329@163.com (S.Z.); qinsheng@just.edu.cn (S.Q.); sunxia8428@163.com (X.S.); xueyangwang@just.edu.cn (X.W.); 2 Key Laboratory of Silkworm and Mulberry Genetic Improvement, Ministry of Agriculture and Rural Affairs, The Sericultural Research Institute, Chinese Academy of Agricultural Sciences, Zhenjiang 212100, China

**Keywords:** AcMNPV, *Bombyx mori*, *BmRRS1*, siRNA

## Abstract

The study of functional genes involved in baculovirus infection is vital for its wide application in pest biocontrol. This study utilized the *Autographa californica* nucleopolyhedrovirus (AcMNPV) and silkworm as models to elucidate the role of BmRRS1, which has been found to exhibit notable differential expression between resistant and susceptible silkworm strains. The results showed that it was evolutionarily conserved in selected species. Among different tissues, it was expressed at the highest level in the gonads, followed by the hemolymph and silk glands; among the different developmental stages, it was the highest in the second instar, followed by the pupae and adults. Moreover, its vital role in suppressing AcMNPV infection was verified by the decreased expression of *lef3* and vp39 protein after overexpression of *BmRRS1* as well as by the increased expression of the viral gene *lef3* and the viral protein vp39 after siRNA treatment against *BmRRS1* expression in BmN cells. Additionally, the direct interaction between BmRRS1 and AcMNPV was detected by the GST pull-down assay. Finally, the homologue of *BmRRS1* in *Spodoptera frugiperda* was found to be involved in larval resistance to AcMNPV. In a word, *BmRRS1* plays a vital role in AcMNPV resistance in silkworms, and this might be related to the direct interaction with AcMNPV. The results of this study provide a potential target for protecting silkworm larvae from virus infection and controlling agricultural and forestry pests.

## 1. Introduction

*Autographa californica* nucleopolyhedrovirus (AcMNPV) is a representative baculovirus species and one of the pathogens infecting the lepidopteran pest *Autographa californica* [1]. Baculovirus is widely used as an insecticide for pest control in agriculture and forestry. AcMNPV can cross-infect more than 30 lepidopteran pests [2], including *Spodoptera frugiperda* [3]. AcMNPV has a wider host domain than other baculoviruses both in vitro and in vivo. Therefore, the research on AcMNPV as an efficient, safe and pollution-free biological insecticide is deepening.

Current studies investigating the interaction between AcMNPV and cells mainly focus on three aspects. First, the studies focus on inhibition of host transcription and translation by AcMNPV. In Sf9 cells infected with AcMNPV, the level of host protein synthesis decreased starting at 6 h to 10 h after infection and ceased completely at 24 h [4]. Second, AcMNPV inhibited host cell apoptosis. For example, baculovirus genes *P35*, *P49* and *IAP* are thought to play a key role in blocking the apoptosis induced by viral infection [5,6]. Third, AcMNPV arrested cell cycle progression. The baculovirus gene *ie-2* expressed early prevents the cell cycle from entering the S phase and thereby regulates gene expression in a variety of cell lines [7]. Although AcMNPV is a model species of baculoviruses and the silkworm is a model insect of Lepidoptera, there are still few studies on the interaction between AcMNPV and the host protein of the silkworm. These studies are crucial for elucidating the mechanism of host resistance to virus infection and its wide application in pest control.

The mulberry silkworm, also known as *Bombyx mori*, is a lepidopteran species belonging to family Bombycidae under phylum Arthropoda. It originated in China and contributed greatly to the development of the silk industry and civilization worldwide [8]. Diseases affecting silkworms are caused by viruses, bacteria, fungi, protozoa, and other organisms. However, viral diseases are the most common and frequent in silkworms. Nuclear polyhedrosis caused by baculoviral infection reduces cocoon production, leading to huge economic losses every year. Infection caused by the nucleopolyhedrovirus (NPV) is usually fatal to insects. Due to the strong infectivity of the virus and its rapid replication in host cells, it is difficult to prevent [5]. At present, there are many studies on the interaction mechanism of NPV or baculovirus with silkworms, but the mechanism of resistance to baculovirus infection in silkworms is still unclear [9,10,11]. The study of some new infection models will be of great help in elucidating the mechanism.

In eukaryotic cells, the ribosome consists of two ribonucleoprotein subunits, the 60S large subunit and the 40S small subunit [12]. In 1999, Tsuno et al. discovered a new regulatory protein for ribosome synthesis in yeast, named as regulator of ribosome biosynthesis 1 (RRS1). RRS1 participates in the maturation of 25SrRNA and the assembly of the 60S subunit during ribosome biosynthesis [13]. Ribosome proteins not only play an important regulatory role in the biosynthesis of ribosomes but also participate in the regulation of multiple activities inside cells, especially cell proliferation and apoptosis [14]. It has been reported that host ribosomal protein S20 and ribosomal protein universal S10 inhibit viral replication by interacting with viral proteins [15,16]. In addition, studies have shown that ribosome biogenesis restricts innate immune responses to virus infection and DNA, ribosome biosynthesis and rRNA accumulation inhibit the replication of human cytomegalovirus (HCMV) [17]. Cao et al. reported that RRS1 binds with RPL11 during apoptosis mediated via the p53 pathway [18]. This provides us with a new idea to explore the mechanism of *BmRRS1* underlying virus infection in silkworms.

In a previous study in our laboratory, RNA-Seq sequencing was performed on the hemolymph of p50 and C108 infected with AcMNPV after 6 h of infection, and several differentially expressed genes related to virus infection were screened [19]. Among them, we focused on *BmRRS1*, a significantly downregulated gene in the sensitive silkworm strain p50, of which the function in AcMNPV infection is still unknown. This study aimed to determine the function of *BmRRS1* in AcMNPV infection with multiple technologies and its potential applied value in pest control (Figure S3).

## 2. Results

### 2.1. Bioinformatics Analysis of BmRRS1

**Figure 1:**
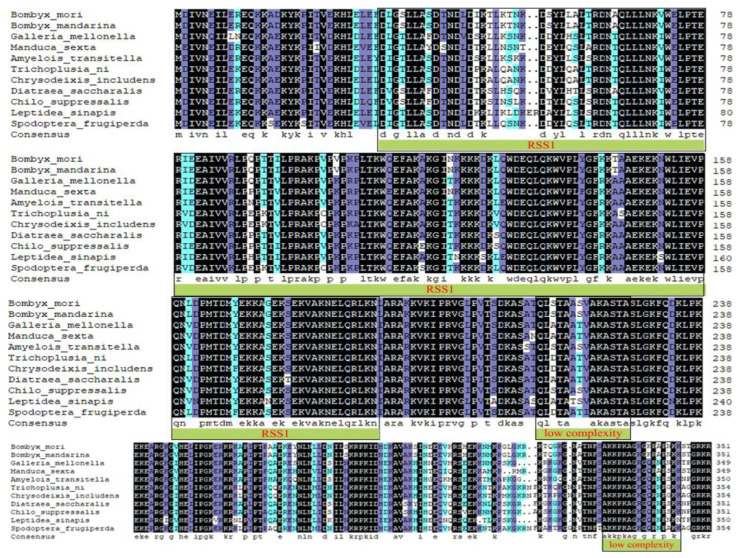
Homologous comparison of amino acid sequences of RRS1 in silkworm and 10 other species. Identical amino acid sequences are marked in black, while different sequences are marked in purple and light blue. The function domains are marked in green.

*BmRRS1* (GenBank ID: XM_004921917.3) contains 1311 bp, with a CDS of 1059 bp encoding 352 amino acid molecules to form a protein. Analysis of the functional domains of BmRRS1 revealed that the functional domain of RRS1 was located at amino acids 28-190. Using SMART prediction, the amino acid sequence of RRS1 was found to carry two low-complexity regions and one functional domain. We obtained homologous nucleotide sequences of other species with BmRRS1 from NCBI and compared the protein homology sequences using DNAMAN 8.0 (Figure 1). The result showed that the amino acid sequence homology of *Bombyx mandarina* was close to 100%.

**Figure 2:**
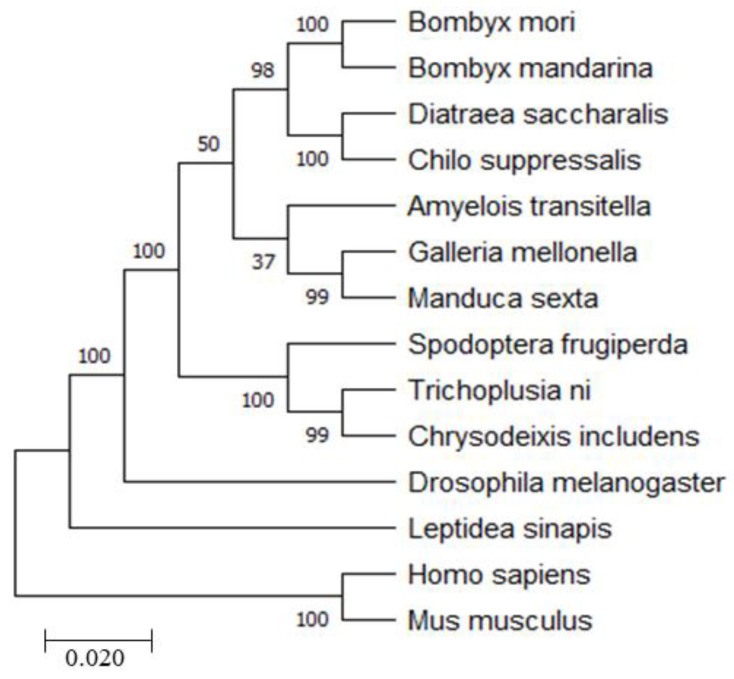
System phylogenetic tree of RRS1. The tree was constructed with pairwise deletion of gaps in MEGA-X. The percentages on the branches indicate bootstrap values from 1000 replicates. The tree is drawn to scale, with branch lengths in the same units as those of the evolutionary distances used to infer the phylogenetic tree. The evolutionary distances were computed using the *p*-distance method and are expressed as units of the number of amino acid differences per site. The analysis involved 14 amino acid sequences. All positions containing gaps and missing data were eliminated.

To further analyze the evolutionary relationships of RRS1 in different species, a phylogenetic tree was created using MEGA-X (Figure 2). The result showed that BmRRS1 was closely related to *B. mandarina*, *Trichoplusia ni*, *Manduca sexta, Spodoptera frugiperda* and *Chrysodeixis includens* but distantly related to *Homo sapiens* and *Mus musculus*, suggesting that RRS1 might be genetically differentiated in different species.

### 2.2. Spatiotemporal Expression Profile of BmRRS1

**Figure 3:**
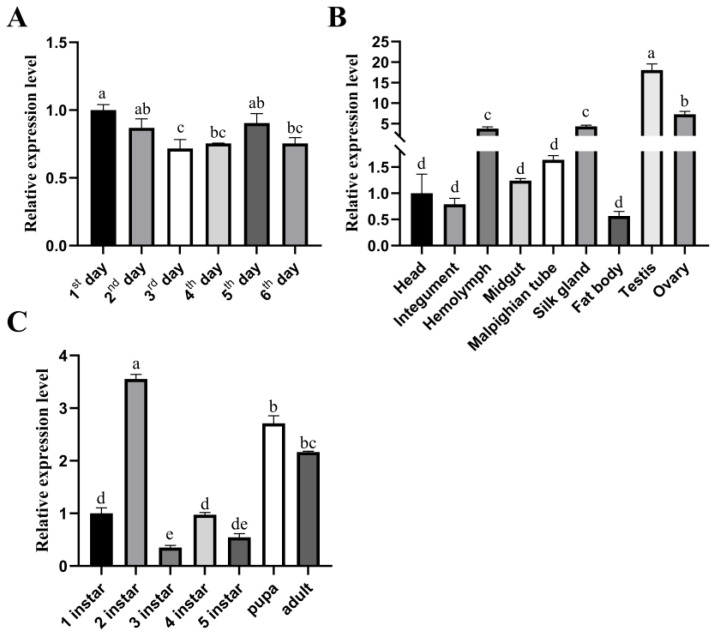
Temporal and spatial expression profile of *BmRRS1* in p50 silkworm. (**A**) Expression of *BmRRS1* during different developmental stages in the egg period. (**B**) Expression of *BmRRS1* in different tissues on the third day of the 5th instar. (**C**) Expression of *BmRRS1* on the first day of different developmental stages. Data were normalized using the reference gene *BmGAPDH*, with the mean ± SD of three independent biological replicates. Statistically significant differences between samples were analyzed using one-way ANOVA followed by the post hoc Tukey’s test. *p* < 0.05 is indicated using different letters a, b, c, d, etc.

To analyze the function of *BmRRS1,* the expression level of *BmRRS1* in different instars and tissues of p50 silkworms was detected by RT-qPCR. The results showed that *BmRRS1* was expressed in 1–6 days of the egg stage (Figure 3A). In different tissues, the expression level was highest in the testis followed by the ovary, hemolymph, and silk gland and the lowest in the fat body (Figure 3B). The expression level of *BmRRS1* was highest in the second instar followed by pupal and adult stages (Figure 3C).

### 2.3. Expression of BmRRS1 in Immune System-Related Tissues of Different Resistant Silkworm Strains

**Figure 4:**
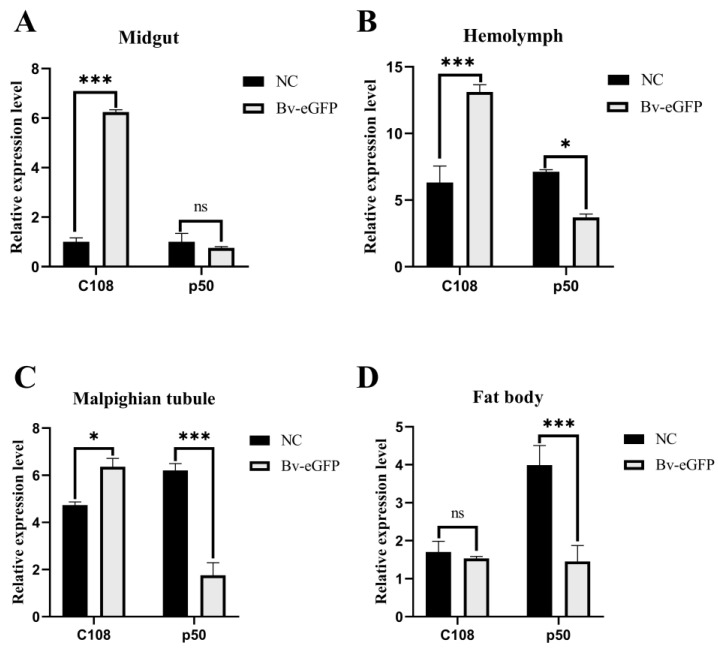
Expression levels of *BmRRS1* in different tissues of domesticated silkworms of sensitive (p50) and resistant (C108) strains after infection with AcMNPV were analyzed using RT-qPCR. Expression levels of *BmRRS1* in (**A**) midgut, (**B**) hemolymph, (**C**) Malpighian tubule and (**D**) fat body at 36 h following AcMNPV infection. Data were normalized using the reference gene *BmGAPDH*, and the mean ± standard deviation was determined from three independent biological replicates. Statistically significant differences between samples were analyzed using one-way ANOVA followed by the post hoc Tukey’s test. Significant differences are indicated by asterisks. ns *p* > 0.05, * *p* < 0.05, *** *p* < 0.001.

To further verify its function, we injected AcMNPV virus into the sensitive strain p50 and the resistant strain C108 (BV-eGFP represents the experimental group). The control group was injected with the same volume of Sf-900^TM^ (NC represents the control group). In a previous study, we found that p50 and C108 showed differences in phenotype and physiological function after infection with AcMNPV [20]. Therefore, four immune-related tissues including the midgut, hemolymph, fat body, and Malpighian tubule were dissected at 36 h after virus infection. To detect whether the infection was successful, the expression of the viral gene *lef3* in the midgut, hemolymph, Malpighian tubule and fat body of p50 and C108 was detected. The results showed that each of the tissues had a significant infection of BV-eGFP, and different tissues had different infection levels (Figure S1), indicating that the samples could be used in the following tests. The RNA was extracted and the expression of *BmRRS1* was analyzed at the transcriptional level using RT-qPCR. The results showed that the expression of *BmRRS1* in the hemolymph, fat body, and Malpighian tubule of the experimental group was significantly lower than in the control group in p50; the expression of *BmRRS1* in the midgut, hemolymph and Malpighian tubule of the experimental group was significantly higher than that in the control group in C108 (Figure 4).

### 2.4. Overexpression of BmRRS1 Inhibited AcMNPV Proliferation in BmN Cells

**Figure 5:**
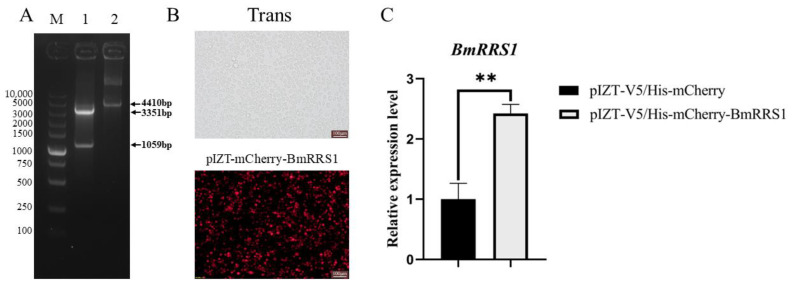
Analysis of *BmRRS1* overexpression in BmN cells. (**A**) Double digestion verification of pIZT/V5-His-mCherry-BmRRS1 overexpression vector. 1. pIZT/V5-His-mCherry-BmRRS1 double enzyme digestion product, 2. pIZT/V5-His-mCherry-BmRRS1, M. Molecular weight of DNA. (**B**) Fluorescence of BmN cells after pIZT/V5-His-mCherry-BmRRS1 transfection (scale: 100 μm). (**C**) Expression of *BmRRS1* in overexpressed group of cells. Data were normalized using the reference gene *BmGAPDH*, and the mean ± standard deviation was determined from three independent biological replicates. Statistically significant differences between samples were analyzed using one-way ANOVA followed by the post hoc Tukey’s test. Significant differences are indicated by asterisks. ** *p* < 0.01.

**Figure 6:**
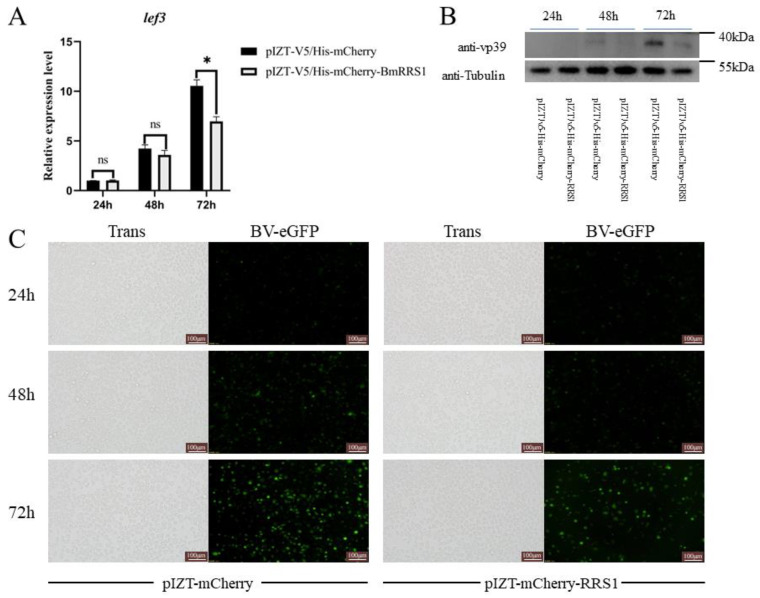
Analysis of the role of overexpressed *BmRRS1* in viral proliferation. (**A**) Viral gene *lef3* expression at different time points after *BmRRS1* overexpression; (**B**) Viral protein vp39 expression at different times after *BmRRS1* overexpression; (**C**) Fluorescence signals emitted by eGFP were detected at 24 h, 48 h and 72 h after infection (scale: 100 μm). Data were normalized using the reference gene *BmGAPDH*, and the mean ± standard deviation was determined from three independent biological replicates. Statistically significant differences between samples were analyzed using one-way ANOVA followed by the post hoc Tukey’s test. Significant differences are indicated by asterisks. ns *p* > 0.05, * *p* < 0.05.

To detect the effect of *BmRRS1* on viral infection, we constructed a transgenic cell line with a pIZT-mCherry-BmRRS1 vector. Red fluorescence was observed via fluorescence microscopy, and the expression level of the *BmRRS1* gene was determined by RT-qPCR. The pIZT-mCherry-BmRRS1 recombinant plasmid was successfully transfected and stably expressed in BmN cells (Figure 5B). The result showed that the expression of *BmRRS1* in the experimental group was significantly up-regulated compared with the control group (Figure 5C). The infection of AcMNPV was detected by fluorescence microscopy and the expression of the AcMNPV late gene *lef3* was analyzed at 24 h, 48 h and 72 h after adding BV-eGFP. The green fluorescence signal of the virus in the experimental group was lower than that in the control group at 48 h and 72 h (Figure 6C). In addition, the expression of *lef3* in the experimental group was lower than in the control group at 72 h (Figure 6A), indicating that *BmRRS1* induced resistance to AcMNPV. Furthermore, based on Western blot analysis, the expression of AcMNPV vp39 protein in the experimental group was lower than in the control group at 72 h after virus infection (Figure 6B). Thus, *BmRRS1* inhibits the proliferation of AcMNPV in silkworms.

### 2.5. Knockdown of BmRRS1 Promoted AcMNPV Proliferation in BmN Cells

**Figure 7:**
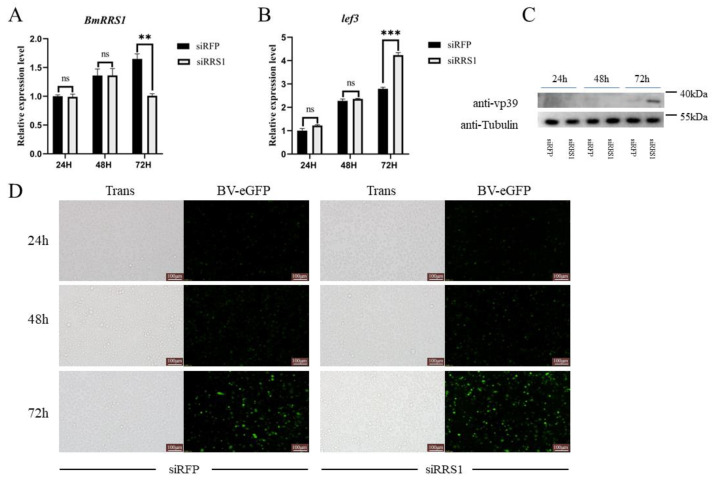
Analysis of the role of *BmRRS1* knockdown in viral proliferation. (**A**). Expression of *BmRRS1* at different time points after siRNA transfection; (**B**). Viral gene *lef3* expression at different times; (**C**). Expression of viral protein vp39 at different time points after *BmRRS1* knockdown; (**D**). Fluorescence signals emitted by eGFP were detected at 24 h, 48 h and 72 h after infection (scale: 100 μm). Data were normalized using the reference gene *BmGAPDH*, and the mean ± standard deviation was determined from three independent biological replicates. Statistically significant differences between samples were analyzed using one-way ANOVA followed by the post hoc Tukey’s test. Significant differences are indicated by asterisks. ns *p* > 0.05, ** *p* < 0.01, *** *p* < 0.001.

To further verify the role of *BmRRS1* in AcMNPV infection of BmN cells, we designed two targets on the functional domain of BmRRS1. The designed synthetic siRNA was based on these two targets. The synthetic siRRS1 was transfected into BmN cells, and the virus containing BV-eGFP was added. AcMNPV infection was detected by fluorescence microscopy at 24 h, 48 h and 72 h after BV-eGFP addition. siRFP was transfected into the control group. After transfection of siRRS1 into BmN cells, the viral fluorescence in the experimental group was increased at 72 h. However, no significant difference was found at 24 h and 48 h (Figure 7D). The expression of *BmRRS1* and *lef3* was analyzed by RT-qPCR. Among three time points, the expression level of *BmRRS1* was just significantly down-regulated after 72 h of siRNA transfection (Figure 7A). At 72 h, the *lef3* expression in the experimental group was significantly higher than in the control group (Figure 7B). Also, the results of Western blot analysis showed that the expression of viral protein vp39 in the experimental group was higher than in the control group at 72 h after viral infection (Figure 7C).

### 2.6. BmRRS1 Interacts with BV-eGFP to Affect Its Infection

**Figure 8:**
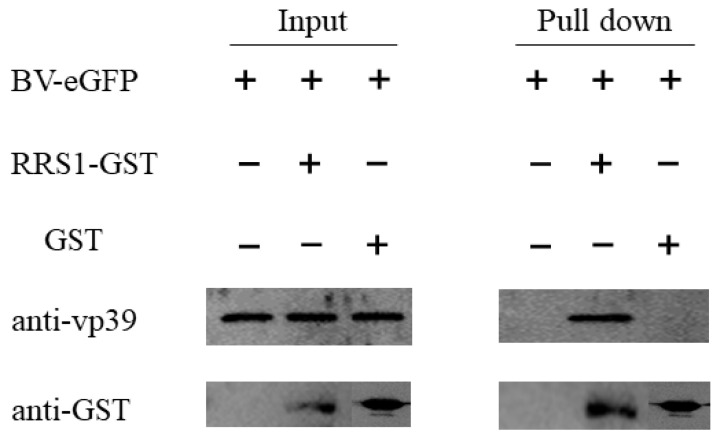
Interaction between BmRRS1 and AcMNPV. GST pull-down of BV-eGFP was performed using purified BmRRS1 labeled with GST and BV-eGFP as bait.

To investigate the mechanism of viral regulation of BmRRS1, we used the GST-tag pull-down assay to determine the interaction between BV-eGFP and BmRRS1. The recombinant plasmid pGEX-4T-1-BmRRS1 was constructed and validated by *BamH* I and *Xhol* I (Figure S2). Then, the purified fusion protein BmRRS1 was used to incubate with BV-eGFP. The results showed that the blank control group (BV-eGFP) did not bind to GST-Beads, and no BV-eGFP was detected in the eluate after washing. However, BV-eGFP could not be detected in the eluent although the negative control could bind to beads. In addition, after incubating with the experimental group of BV-eGFP and BmRRS1 recombinant protein, BV-eGFP was found to bind to the BmRRS1 protein in the eluate (Figure 8). Therefore, we assumed that BmRRS1 interacts with AcMNPV to affect virus infection.

### 2.7. Knockdown of SfRRS1 Decreases the Survival Rate of Spodoptera frugiperda to AcMNPV

**Figure 9:**
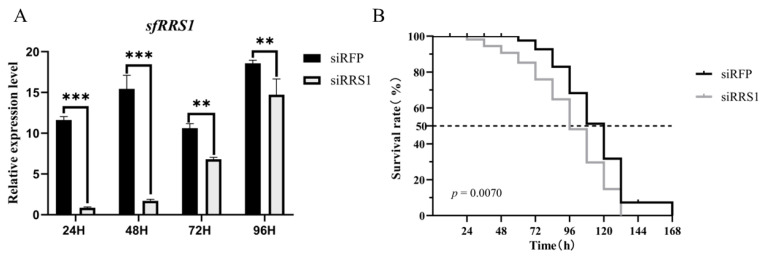
Effect of *SfRRS1* on the survival rate of *Spodoptera frugiperda* larvae after infection with AcMNPV. (**A**) The analysis of expression level of *SfRRS1* after siRRS1 treatment. (**B**) The survival rate of *Spodoptera frugiperda* larvae at different times after injection of BV-eGFP. The dashed line corresponds to a survival rate of 50 %. Data were normalized using the reference gene *SfGAPDH*, and the mean ± standard deviation was determined from three independent biological replicates. Statistically significant differences between samples were analyzed using one-way ANOVA followed by the post hoc Tukey’s test. Significant differences are indicated by asterisks. ** *p* < 0.01, *** *p* < 0.001.

To further verify the function of *BmRRS1* in pest biological control, bioassays were performed in *Spodoptera frugiperda* to evaluate its response to AcMNPV infection after knockdown of *SfRRS1*. The results showed that after injection of siRRS1, the expression of *SfRRS1* in *Spodoptera frugiperda* larvae was significantly downregulated (Figure 9A), and the survival rate of *Spodoptera frugiperda* larvae was significantly decreased after AcMNPV infection (Figure 9B). The results showed that the gene had potential application value in biological control of pests.

## 3. Discussion

The use of baculoviruses as biological insecticides in agricultural pest control is widely practiced. For instance, AgMNPV has been utilized in Brazil to control soybean looper larvae, whereas CpGV is used in North America and Europe to prevent codling moth infestation in apple orchards. HearNPV is used in China to combat cotton bollworms [21,22,23]. AcMNPV, which has a broad host range and infects more than 30 lepidopteran pests, is the most extensively researched baculovirus. As a novel bioinsecticide, it has been utilized in the management of crop pests and diseases. Fan et al. have demonstrated that a recombinant baculovirus (AcMNPV-BmKIT-Chi) showed superior insecticidal efficacy [24]. In addition, it was shown that some silkworm species can be infected by AcMNPV when recombinant AcMNPV was injected into silkworm by puncture. We found that the p50 silkworm was sensitive to AcMNPV, while the C108 silkworm was resistant to AcMNPV [17]. However, the generation of pest resistance will lead to reduced control effects. The two silkworm strains, p50 and C108, provide us with a good research model. Therefore, exploring the mechanism of resistance has been investigated to provide theoretical support for the mechanism of virus infection in insects.

RRS1, as a regulator of ribosome synthesis, binds with ribosome production factor 2 (RPF2) during 25S rRNA maturation and 60S ribosomal large subunit assembly [25,26,27]. Based on a comparison of protein homology sequences of BmRRS1 in different species, its amino acid sequence was strongly similar to that of other species and carried a conserved functional protein (Figure 1). The phylogenetic tree analysis revealed conservative evolution of BmRRS1 (Figure 2). It has been found that RRS1 (together with RPF2) binds with RPL11 and anchors it in the nucleolus during apoptosis mediated via the p53 pathway [18]. In this study, *BmRRS1* was identified in p50, a silkworm strain that is susceptible to AcMNPV. Its expression level varied significantly before and after AcMNPV infection. Therefore, we hypothesized that RRS1 may be involved in viral infection.

The stable expression of *BmRRS1* in the egg stage indicates its critical role in the development of silkworms (Figure 3A). RRS1 acts as a ribosomal protein synthesis regulator to promote ribosomal protein synthesis and participates in cell proliferation and apoptosis [28]. It is well known that the silk gland is a place for silk protein synthesis and storage [29]. The nuclei of silk gland cells begin to branch at the second instar [30,31]; the high expression of *BmRRS1* may be related to this. (Figure 3C). Its high expression in the pupal and adult stages indicates that the gene may be regulated by ecdysone. (Figure 3C). In addition, the high expression in the testis and ovary indicates that it is involved in the development of gonads (Figure 3B). Using RT-qPCR, we analyzed the expression of *BmRRS1* in different resistant silkworm strains after AcMNPV infection. The results showed that the expression of *BmRRS1* in the hemolymph, fat body and Malpighian tubule of the experimental group was significantly lower than in the control group in the sensitive strain p50 (Figure 4). We speculate that *BmRRS1* is involved in the infection of silkworm by AcMNPV, but its mechanism of action is still unclear. To elucidate the underlying mechanism, we conducted overexpression and interference experiments at the cellular level. Compared with the control group, the expression of the virus gene *lef3* was decreased after the overexpression of *BmRRS1* (Figure 6A). The green fluorescence signal of the virus in the experimental group was lower than that in the control group at 48 h and 72 h (Figure 6C). In addition, at 72 h, the expression of viral protein vp39 in the experimental group was significantly lower than in the control group after virus infection, based on the results of Western blot analysis (Figure 6B). When *BmRRS1* was knocked down in BmN cells, the expression level of both the viral gene *lef3* and the viral protein vp39 were increased (Figure 7), indicating that *BmRRS1* inhibited the proliferation of AcMNPV in silkworms.

Studies have shown that ribosomal proteins (RPs) play an antiviral role mainly in two ways, one of them is that ribosomal proteins directly inhibit the transcription or translation of viral proteins by interacting with viruses [32,33]. To explore the mechanism of *BmRRS1* inhibiting AcMNPV infection in silkworm, BmRRS1-GST fusion protein was expressed and purified and used to analyze the interaction with vp39. The results revealed that GST and vp39 could be detected using BmRRS1-GST as bait, indicating the interaction between BmRRS1 and BV-eGFP in vitro (Figure 8).

Previous studies have confirmed that BmRRS1 is localized in the nucleus [28]. Budded virions are present in both the nucleus and cytoplasm during baculovirus infection of host cells [34]. Therefore, we hypothesized that BmRRS1 binds to a protein of the virus, thereby inhibiting the transcription or translation of viral proteins. However, the specific binding proteins of BmRRS1 to AcMNPV are still unknown, suggesting the need for further studies in the future. This comprehensively suggests that the interaction between BmRRS1 and AcMNPV regulates the infection of domestic silkworms by the virus.

In conclusion, our results indicate that increasing the expression of *BmRRS1* can enhance the resistance of silkworms to baculovirus. In addition, the bioassay of *Spodoptera frugiperda* confirmed that inhibiting the expression of *RRS1* can result in a better insecticidal effect. RRS1 can serve as an important potential target for pest control.

## 4. Materials and Methods

### 4.1. Silkworms and AcMNPV

*Bombyx mori* strains p50 and C108 were obtained from the Key Laboratory of Sericulture, School of Biotechnology, Jiangsu University of Science and Technology; p50 was sensitive to AcMNPV and C108 was resistant. Fresh mulberry leaves were used to feed the two strains, which were incubated at a temperature of 26 ± 1 °C and a relative humidity of 75 ± 5% and under a constant 12 h light/12 h dark photoperiod. The temperature was adjusted to 24 ± 1 °C at the fourth and fifth instar stages, and conditions such as relative humidity remained unchanged [20].

The BmN cell line was derived from the silkworm ovary, and the Sf9 cell line was derived from the ovary of *Spodoptera frugiperda*. Both cell lines were cultured and cryopreserved in our laboratory. The medium was changed or passaged when the cell density reached 70–80%. The number of cell passages used in this experiment was within fewer generations.

AcMNPV virus carrying an enhanced green fluorescent protein tag (BV-eGFP) was stored in the Key Laboratory of Sericulture, School of Biotechnology, Jiangsu University of Science and Technology. The polyhedrin promoter was deleted, and a *Bombyx mori* cytoplasmic actin-3 promoter-driven eGFP cassette was inserted into the MCS of the AcMNPV bacmid [35]. Viruses were propagated in Sf9 cells, collected by centrifuge, observed by fluorescence microscope and stored at 4 °C. The method for determining BV-eGFP titers has been described previously [36].

### 4.2. Sample Preparation

To explore the expression pattern of BmRRS1, the eggs of p50-strain silkworms from 1–6 days and whole silkworms on the first day of each instar were collected. The different tissues of p50 silkworms on the third day of the fifth instar were dissected. Each silkworm was injected with 1.0 µL medium containing BV-eGFP (1.0 × 10^8^ pfu/mL) on the first day of the fifth instar to analyze the immune response. The control group was treated with the same volume of the medium. The silkworm tissues (midgut, hemolymph, fat body and Malpighian tubule) were collected at 36 h after injection. All samples were collected on ice in a sterile 1.5 mL centrifuge tube. The hemolymph was collected in a centrifuge tube containing a small amount of thiourea to prevent oxidation, and each sample volume was at least 500 μL. Other tissues were rinsed in pre-cooled normal saline to ensure that the samples were free of impurities. The samples were then pulverized in the presence of liquid nitrogen and stored at −80 °C. Each group of 5 samples was pooled together to eliminate experimental errors caused by individual genetic differences. The experiment was repeated three times.

### 4.3. RNA Extraction and cDNA Synthesis

Total RNA was extracted from 10 mg tissue powder or 500 μL hemolymph by mixing with 500 μL RNAiso Plus (TaKaRa, Dalian, China) according to manufacturer’s instructions. The volume of chloroform was 200 μL, the volume of isopropanol was 250 μL, and the volume of 75% ethanol was 500 μL. The RNA precipitate was dissolved in 50 μL of DEPC water. The NanoDrop 2000 spectrophotometer (Thermo Scientific, Waltham, MA, USA) was used to detect the absorbance ratio of 260/280 and concentration of RNA, and its quality was checked by 1% agarose gel electrophoresis. The first-strand cDNA was synthesized with 1.0 μg RNA using the PrimeScript^TM^ RT reagent kit (TaKaRa, Dalian, China), according to the manufacturers’ instructions. The cDNA was stored at −20 °C.

### 4.4. Bioinformatic Analyses

The *BmRRS1* nucleic acid sequence (XM_004921917.3) and its homologous sequences in other species were retrieved from NCBI (http://www.ncbi.nlm.nih.gov/ (accessed on 2 October 2022)). We selected the GenBank database and entered the gene and species name in the search box. The target gene was selected and its nucleotide and protein sequences were obtained. Homologous sequences in other species were obtained by nucleotide blast or protein blast (Table S1). The functional domains of BmRRS1 were predicted using SMART online (http://smart.embl-heidelberg.de/ (accessed on 5 October 2022)). Domain prediction was performed by either a Uniprot/Ensembl sequence identifier (ID)/accession number (ACC) or the protein sequence itself. We selected outlier homologues and homologues of known structure, PFAM domains, signal peptides and internal repeats for smart sequencing. The homology of the BmRRS1 nucleotide sequence was analyzed with DNAMAN 8.0 software. A neighbor-joining tree was generated using MEGA-X, with 1000 bootstrap replications and an optimal DNA/protein model of LG + G.

### 4.5. Cell Culture and Transfection

The culture medium of BmN cells consisted of TC-100 (PanReac AppliChem, Darmstade, Germany, pH 6.2), 10% fetal bovine serum (FBS) (Thermo Fisher Scientific, New York, NY, USA) and 1% antibiotics (penicillin and streptomycin, AppliChem, Germany). The culture medium of Sf9 cells was Sf-900^TM^ (Thermo Fisher Scientific, New York, NY, USA). The culture temperature was 27 °C. Neofect^TM^ DNA transfection reagent (A Genome Technologies Corporation, Beijing, China) was used to transfect the overexpression vector and siRNAs, according to the manufacturer’s instructions. Each 60 mm dish was transfected with 4.0 µg of plasmid or siRNA. The plasmid or siRNA was first mixed with 200 µL FBS-free TC-100, followed by 4.0 µL Neofect^TM^ transfection reagent and then left to stand for 15 to 30 min. The stable cell strain was screened by zeocin (Invitrogen, Carlsbad, CA, USA).

### 4.6. Construction of pIZT-mCherry-BmRRS1 Overexpression Vector

The primers for amplifying the coding sequence of *BmRRS1* were designed according to the primers designed by NCBI’s Primer-BLAST (*BmRRS1-1* shown in Table 1; the underline represents a restriction enzyme cutting site). The sequence was derived from the cDNA of BmN cells, and the purified product was cloned with pMD-19T vector. All sequencing steps were performed at Zhejiang SUNYA Biotechnology (Zhejiang, China). The correct sequence and pIZT/V5-His-mCherry overexpression vector were ligated with T4 DNA ligase (TaKaRa, Dalian, China) and verified by double digestion with *Kpn* I and *Xba* I (TaKaRa, Dalian, China).

### 4.7. Synthesis of siRNA

To knock down *BmRRS1* expression levels, targets located in the functional domain of *BmRRS1* were designed using an online website (https://www.genscript.com/tools/sirna-target-finder (accessed on 18 October 2022)). The target sequence was synthesized by SUNYA Biotechnology (Zhejiang, China). The specific sequence information is shown in Table 2. The siRNAs were synthesized using an in vitro transcription T7 kit (TaKaRa, Dalian, China) according to the manufacturer’s instructions. The synthesized siRNA was purified with Acidic Phenol–Chloroform–Isoamyl Alcohol (25:24:1, Shaoxinbio, Shanghai, China). The absorbance at 260/280 and concentration were detected by the NanoDrop 2000 spectrophotometer (Thermo Scientific, New York, NY, USA). The quality of the synthesized siRNA was detected by 3% agarose gel electrophoresis under 160 V for 10 min. Transfection into BmN cells was performed as described in Section 4.5, thereby reducing the expression of *BmRRS1*. Similarly, two siRNAs targeting the functional domain of SfRRS1 were synthesized. The specific sequence information is shown in Table S2. The remaining siRNAs were stored in a refrigerator at −80 °C.

### 4.8. Construction of pGEX-4T-1-BmRRS1 Overexpression Vector

The overexpression vector pGEX-4T-1-BmRRS1 was constructed to induce the expression of BmRRS1 protein in BL21 cells. Primers for the *BmRRS1* coding sequence were designed using NCBI’s Primer-BLAST. BmN cell cDNA was used as a template for amplification (*BmRRS1-2* in Table 1; the underline represents the *BamH*I and *Xhol*I restriction sites, respectively). The specific construction steps were the same as described in Section 4.6, including the restriction sites *BamH* I and *Xhol* I.

### 4.9. Real-Time Quantitative PCR (RT-qPCR)

The gene relative expression in the sample’s cDNA was detected by RT-qPCR using a NovoStart^®^ SYBR qPCR SuperMix Plus kit (Novoprotein, Suzhou, China). It comprised 1.0 μL of template, 5.0 μL of 2× NovoStart^®^ SYBR qPCR SuperMix Plus (Novoprotein, China), 0.5 μL of each primer (10 μM) and 3.0 μL of double-distilled water (ddH_2_O). The qPCR reaction was carried out in the LightCycler^®^ 96 system (Roche, Basel, Switzerland). The thermal cycling program consisted of pre-denaturation at 95 °C for 5 min, 40 cycles at 95 °C for 15 s and 60 °C for 30 s. A melt curve analysis, performed at the end of the PCR cycles, displayed a single sharp peak from 80 to 85 °C. After obtaining the Cq value of the sample, the relative expression level was calculated by the 2^−ΔΔCT^ method. The statistical difference between the three biological replicates was calculated via one-way analysis of variance. GraphPad Prism was used to plot the graph. Glyceraldehyde-3-phosphate dehydrogenase (*BmGAPDH*) was used as an internal reference gene, and late expression factor 3 (*lef3*) was used to detect AcMNPV. The primers are shown in Table 2.

### 4.10. Western Blot

Cells in each sample were suspended by adding 200 μL RIPA lysate (strong) buffer (Beyotime Biotechnology, Shanghai, China) and 2 μL phenylmethyl-sulfonyl fluoride (PMSF), centrifuged at 15,000× *g* for 10 min at 4 °C. We transferred the supernatant to a new centrifuge tube and SDS-PAGE loading buffer was added, followed by incubation in a water bath at 98 °C for 10 min. The proteins were separated by SDS-PAGE and transferred to PVDF membranes. Mouse anti-GST-tag monoclonal antibody (1:4000, TRAN TRANSGEN BIOTECH, Beijing, China), rabbit polyclonal antibody against vp39 (1:500, HUABIO, Zhejiang, China) and mouse monoclonal antibody against β-Tubulin (1:4000, Bioss ANTIBODIES, Beijing, China) were used. Horseradish peroxidase (HRP)-conjugated anti-mouse IgG and rabbit IgG (1:5000, ABclonal Technology, Wuhan, China) were used as secondary antibodies. PVDF membranes were visualized using the ChemiScope series 3600 imager (CliNX, Shanghai, China) to detect protein signals.

### 4.11. GST-Tag Pull-Down

The GST-tag pull-down method was used to verify the interaction between BmRRS1 fusion protein and BV-eGFP. The constructed PGEX-4T-1-BmRRS1 was transformed into *Escherichia coli* BL21, and different final concentrations of IPTG (0,0.2 mM, 0.4 mM, 0.8 mM, 1 mM) were designed to induce the expression of BmRRS1 protein at 37 °C and 16 °C. The optimal final concentration and temperature were screened and the protein solubility was analyzed by ultrasonic crushing. The bacteria induced by IPTG were collected and the BmRRS1 protein was purified by pGEX-4T-1 prokaryotic expression vector and GST agarose beads (QIAGEN, Germany). The protein concentration was obtained by SDS-PAGE, BSA quantification and Image J 1.8.0 software analysis. BmRRS1 (300 ng) and BV-eGFP (viral titer 1 × 10^8^ pfu/mL) were incubated for 24 h at 4 °C on a shaker, followed by the addition of agarose beads to bind with the BmRRS1 fusion protein for 4 h at 4 °C. After washing three times with TNET buffer, proteins bound to GST agarose beads were eluted with elution buffer and detected by Western blotting. Fusion proteins were separated by SDS-PAGE and analyzed with rabbit anti-VP39 and mouse anti-GST. The pGEX-4T-1 vector was induced and purified according to the above method, and the purified GST protein was used as a negative control.

### 4.12. Bioassays for Spodoptera frugiperda to AcMNPV

*Spodoptera frugiperda* were obtained from the Key Laboratory of Sericulture, School of Biotechnology, Jiangsu University of Science and Technology. The larvae were fed an artificial diet as suggested by Li et al. and maintained at 27 ± 1 °C, 70 ± 5% relative humidity, with a 8:16 h scoto/photophase [37].

To detect the resistance level of *Spodoptera frugiperda* larvae to AcMNPV after knocking down the expression of *SfRRS1* (Gene Bank ID: XM_035602499.2), a total of 300 larvae on the first day of the second instar were divided into six groups. Each larva in the three treatment groups was injected with 1.0 μL of siRRS1 (1.0 μg/μL), and the other three groups were injected with 1.0 μL of siRFP (1.0 μg/μL) as control. All larvae were injected with BV-eGFP (1.0 × 10^8^ pfu/mL) after injecting siRRS1 or siRFP. The number of dead larvae after feeding AcMNPV was counted every 12 h.

### 4.13. Statistical Analysis

SPSS Statistics 20 software (IBM, Endicott, New York, NY, USA) was used to evaluate the differences between samples using one-way analysis of variance. The Tukey’s test was performed to detect the significance of the difference between the two data sets that followed the normal distribution. For data that did not meet the normality requirement, the Kruskal–Wallis test was used. *p* < 0.05 was considered statistically significant. GraphPad Prism 8.0.1 software (GraphPad software, San Diego, CA, USA) was used to perform statistical analysis and mapping.

## Figures and Tables

**Table 1:** List of primers used in this study.

Primer Names	Forward Primer (5′-3′)	Reverse Primer (5′-3′)
BmRRS1-1	GGGGTACCATGGATATTGTAAATGAGATATTAGA	GCTCTAGATCTCCTTTTCCGTCCAGTA
BmRRS1-2	CGGGATCCATGGATATTGTAAATGAGATATTAGAAAGGG	CCCTCGAGCTATCTCCTTTTCCGTCCAG
BmRRS1-3	AACGCTGAAGACAAACAAAGATTCA	CGAACTACTATGGCCTCTTCGAT
BmGAPDH	CCGCGTCCCTGTTGCTAAT	CTGCCTCCTTGACCTTTTGC
Lef3	CAAACGCGTTGCTTCGTACA	TGCTCGAGTCGGAAGAGGTA
SfRRS1-1	AGCTTGATATTGGCACATTACTAGCTT	CTCTGTAGGAAGCTCCCATATCTTGTTT
SfGAPDH	AGAAGACTGTTGACGGACC	AGGAATGACTTTGCCGAC

**Table 2:** The list of primer sequences used to synthesize siRNA.

Primer Names	Sequences (5′-3′)
BmRRS1-1 Olig-1	GATCACTAATACGACTCACTATAGGGCCCGTGACAATGCTCAGCTACTACTTT
BmRRS1-1 Olig-2	AAAGTAGTAGCTGAGCATTGTCACGGGCCCTATAGTGAGTCGTATTAGTGATC
BmRRS1-1 Olig-3	AACCCGTGACAATGCTCAGCTACTACTCCCTATAGTGAGTCGTATTAGTGATC
BmRRS1-1 Olig-4	GATCACTAATACGACTCACTATAGGGAGTAGTAGCTGAGCATTGTCACGGGTT
BmRRS1-2 Olig-1	GATCACTAATACGACTCACTATAGGGTAAAGTATGGGAGCTTCCCACTGAATT
BmRRS1-2 Olig-2	AATTCAGTGGGAAGCTCCCATACTTTACCCTATAGTGAGTCGTATTAGTGATC
BmRRS1-2 Olig-3	AATAAAGTATGGGAGCTTCCCACTGAACCCTATAGTGAGTCGTATTAGTGATC
BmRRS1-2 Olig-4	GATCACTAATACGACTCACTATAGGGTTCAGTGGGAAGCTCCCATACTTTATT
RFP-Olig-1	GATCACTAATACGACTCACTATAGGGGCACCCAGACCATGAGAATTT
RFP-Olig-2	AAATTCTCATGGTCTGGGTGCCCCTATAGTGAGTCGTATTAGTGATC
RFP-Olig-3	AAGCACCCAGACCATGAGAATCCCTATAGTGAGTCGTATTAGTGATC
RFP-Olig-4	GA TCACTAATACGACTCACTATAGGGATTCTCATGGTCTGGGTGCTT

## Data Availability

The data presented in this study are available in Supplementary Materials. The RNA-seq raw data were deposited to NCBI SRA with the accession numbers SRR25700523 to SRR25700534 (https://www.ncbi.nlm.nih.gov/sra, accessed on 19 August 2023).

## Supplementary Materials

The following supporting information can be downloaded at: https://www.mdpi.com/article/10.3390/ijms25010306/s1.
