# Supplementary material for: BmRRS1 Protein Inhibits the Proliferation of Baculovirus Autographa californica Nucleopolyhedrovirus in Silkworm, Bombyx mori
Source: Int J Mol Sci. 2023 Dec 25;25(1):306. doi: 10.3390/ijms25010306 (PMC10779178; doi:10.3390/ijms25010306)
Supplement: Supplementary file 1 [file ijms-25-00306-s001.zip › ijms-2751504-supplementary.pdf]

International Journal of *Molecular Sciences*

MDPI

Table S1. Homologous of BmRRS1 in other species

Species	GenBank ID
Bombyx mori	XM_004921917.3
Bombyx mandarina	XM_028188077.1
chilo suppressalis	CAH0398028.1
diatraea saccharalis	CAG9786334.1
galleria mellonella	XM_026895936.3
amyelois transitella	XM_013344156.1
manduca sexta	XM_030173131.2
trichoplusia ni	XM_026869436.1
leptidea sinapis	XM_050814772.1
chrysodeixis includens	CAH0579211.1
Homo sapiens	NM_015169.4
Mus musculus	NM_021511.2
Drosophila melanogaster	NM_168144.3

Table S2. The list of primer sequences used to synthesize siRNA

Primer Names	Sequences (5′-3′)
SfRRS1-1 Olig-1	GATCACTAATACGACTCACTATAGGGCGGACACAAATGATTTGGATACAAATT
SfRRS1-1 Olig-2	AATTTGTATCCAAATCATTTGTGTCCGCCCTATAGTGAGTCGTATTAGTGATC
SfRRS1-1 Olig-3	AACGGACACAAATGATTTGGATACAAACCCTATAGTGAGTCGTATTAGTGATC
SfRRS1-1 Olig-4	GATCACTAATACGACTCACTATAGGGTTTGTATCCAAATCATTTGTGTCCGTT
SfRRS1-2 Olig-1	GATCACTAATACGACTCACTATAGGGCGCGTGACAACACACAGTTACTATTTT
SfRRS1-2 Olig-2	AAAATAGTAACTGTGTGTTGTCACGCGCCCTATAGTGAGTCGTATTAGTGATC
SfRRS1-2 Olig-3	AACGCGTGACAACACACAGTTACTATTCCCTATAGTGAGTCGTATTAGTGATC
SfRRS1-2 Olig-4	GATCACTAATACGACTCACTATAGGGAATAGTAACTGTGTGTTGTCACGCGTT

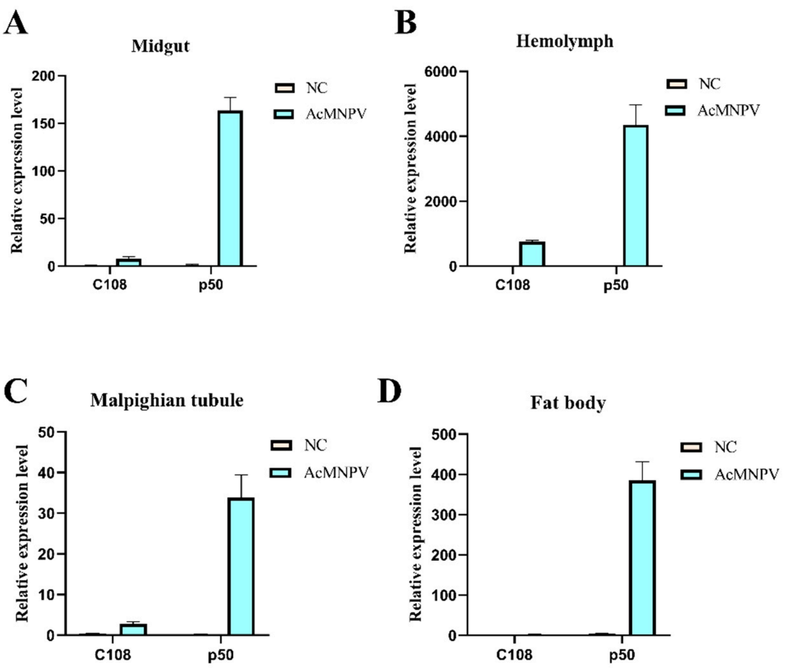

Figure S1. Expression levels of *lef3* in different tissues of domesticated silkworms of sensitive and resistant strains after infection with AcMNPV were analysed using RT-qPCR. Expression levels of *lef3* in (A) midgut, (B) hemolymph, (C) malpighian tubule, and (D) fat body at 36 h following AcMNPV infection. Data were normalised using the reference gene *BmGAPDH*, and the mean ± standard deviation was determined from three independent biological replicates.

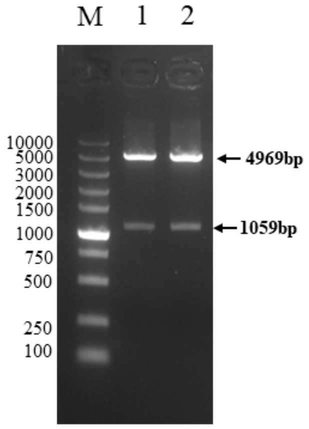

Figure S2. Double digestion verification of pGEX-4T-1-BmRRS1 Overexpression Vector 1,2. pGEX-4T-1-BmRRS1 double enzyme digestion product, M. Molecular weight of DNA.

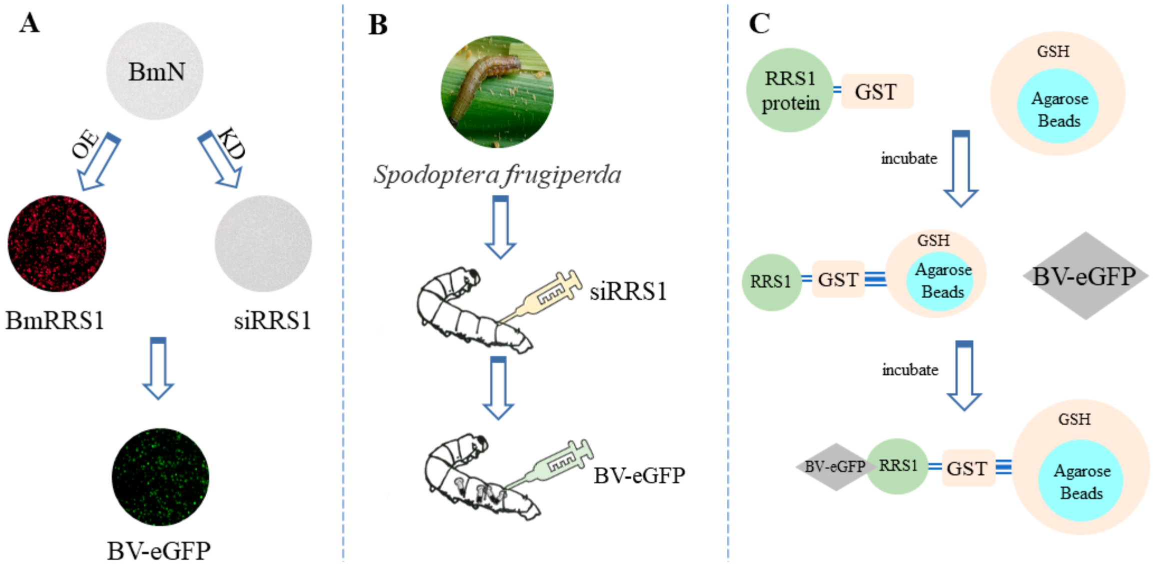

Figure S3. Experimental design. (A) Overexpression or knockdown of *BmRRS1* in BmN cells, (B) Knockdown of *SfRRS1* in Spodoptera frugiperda larvae, (C) GST-taq pull-down assay.
